# AAV-Mediated CRISPRi and RNAi Based Gene Silencing in Mouse Hippocampal Neurons

**DOI:** 10.3390/cells10020324

**Published:** 2021-02-04

**Authors:** Matthias Deutsch, Anne Günther, Rodrigo Lerchundi, Christine R. Rose, Sabine Balfanz, Arnd Baumann

**Affiliations:** 1 Forschungszentrum Jülich, Institute of Biological Information Processing, IBI-1, Leo-Brandt-Straße, 52428 Jülich, Germany; mdeutsch@UCSD.EDU (M.D.); s.balfanz@fz-juelich.de (S.B.); 2 Department of Biology, University of California, San Diego, La Jolla, CA 92083, USA; 3 Center for Molecular Neurobiology Hamburg, University Medical Center Hamburg-Eppendorf, Falkenried 94, 20251 Hamburg, Germany; anne.guenther@zmnh.uni-hamburg.de; 4 Institute of Neurobiology, Heinrich Heine University Düsseldorf, Universitätsstraße 1, 40225 Düsseldorf, Germany; rodrigo.lerchundi.monje@uni-duesseldorf.de (R.L.); Rose@uni-duesseldorf.de (C.R.R.)

**Keywords:** hyperpolarization-activated and cyclic nucleotide-gated ion channel (HCN-channel), immunocytochemistry, knock-down, pacemaker channel, patch-clamp, signaling, transcript quantification, viral transduction

## Abstract

Uncovering the physiological role of individual proteins that are part of the intricate process of cellular signaling is often a complex and challenging task. A straightforward strategy of studying a protein’s function is by manipulating the expression rate of its gene. In recent years, the Clustered Regularly Interspaced Short Palindromic Repeat (CRISPR)/Cas9-based technology was established as a powerful gene-editing tool for generating sequence specific changes in proliferating cells. However, obtaining homogeneous populations of transgenic post-mitotic neurons by CRISPR/Cas9 turned out to be challenging. These constraints can be partially overcome by CRISPR interference (CRISPRi), which mediates the inhibition of gene expression by competing with the transcription machinery for promoter binding and, thus, transcription initiation. Notably, CRISPR/Cas is only one of several described approaches for the manipulation of gene expression. Here, we targeted neurons with recombinant Adeno-associated viruses to induce either CRISPRi or RNA interference (RNAi), a well-established method for impairing *de novo* protein biosynthesis by using cellular regulatory mechanisms that induce the degradation of pre-existing mRNA. We specifically targeted hyperpolarization-activated and cyclic nucleotide-gated (HCN) channels, which are widely expressed in neuronal tissues and play essential physiological roles in maintaining biophysical characteristics in neurons. Both of the strategies reduced the expression levels of three HCN isoforms (HCN1, 2, and 4) with high specificity. Furthermore, detailed analysis revealed that the knock-down of just a single HCN isoform (HCN4) in hippocampal neurons did not affect basic electrical parameters of transduced neurons, whereas substantial changes emerged in HCN-current specific properties.

## 1. Introduction

Information processing in the nervous system relies on the coordinated activity of electrical and chemical signals. At the level of individual neurons as well as in neuronal networks, the depolarization of the membrane voltage leads to the generation of action potentials, which finally cause the release of transmitters that transduce the signal to downstream target cells. Many ion channels that actively contribute to the electrical properties of neurons have been intensively studied by electrophysiological, biochemical, pharmacological, as well as molecular biological methods (for reviews see: [1,2,3,4]). One gene family has been uncovered encoding ion channels with exceptional activation properties, i.e., hyperpolarization-activated and cyclic nucleotide-gated (HCN) channels (for reviews, see: [5,6]). These channels are referred to as ‘pacemaker’ channels, since they are known to largely control the repetitive beating of the heart and also to contribute to rhythmic neuronal activity [7].

In mammals, four genes have been identified encoding HCN channel subunits, with HCN1, 2, and 4 being predominantly expressed in neural tissue [8]. The subunits can form homomeric as well as heteromeric functional ion channels [9]. Thus, the electrical properties of HCN channels are determined by their subunit composition, stoichiometry, and, in addition, can further be modified by accessory subunits, e.g., TRIP8b [10,11,12] as well as by cyclic nucleotides [13]. A characteristic feature of HCN channels setting them apart from the vast majority of voltage-gated ion channels is their activation upon membrane hyperpolarization, rather than depolarization. Under physiological conditions, HCN channel opening leads to an influx of Na^+^ ions, thereby driving the membrane potential back towards the resting membrane potential or to even more positive values, eventually evoking an action potential. Beyond their pacemaking properties, HCN channels contribute to various neural processes, including the dampening of dendritic excitability and regulating transmitter release from the presynaptic terminal [14].

Notably, while HCN channel function has been well described over the years, most physiological, pharmacological, and electrophysiological studies tend to focus on the HCN channel-mediated Ih current as a whole without taking the distinct biophysical properties and specific expression patterns of the four isoforms into account; despite many molecular studies having previously shown that the individual isoforms play distinct, sometimes even complementary roles in the modulation of biophysical properties of neurons [15].

Strategies are required to specifically interfere with HCN channel subunit expression in order to elaborate on the contribution of individual HCN channel subunits to neuronal network and systemic functions because there is a lack of HCN subunit-specific blockers. Here, we evaluated and used two gene-expression interfering tools, i.e., RNAi and CRISPRi, aiming to reduce *hcn* gene expression in post-mitotic neurons. We applied recombinant Adeno-associated viruses (rAAVs) [16] as gene ferries that encoded (1) either *hcn1*-, *2*-, or *4*-directed shRNA molecules or (2) an enzymatic inactive form of *Staphylococcus aureus* Cas9 (dSaCas9) [17], which binds to the promoter regions of the different *hcn* genes that are assisted by specific guide RNAs (sgRNA). The efficacy and specificity of both strategies was examined by qRT-PCR analysis of *hcn* transcript levels.

We found a reduction of the *hcn* transcript levels after treating samples with shRNA as well as sgRNA/dCas9, but not with control constructs. A reduction of *hcn* transcript levels was more pronounced with shRNA-encoding constructs in comparison to sgRNA/dCas9-encoding constructs. To this end, using shRNA encoding rAAVs allowed for us to specifically and efficiently interfere with gene expression in both primary neuronal cultures and organotypic slice cultures. By reducing the expression of the HCN4 channel subunit, we could determine its role in modulating single cell HCN-current related electrophysiological properties in the pyramidal neurons of the hippocampus.

## 2. Materials and Methods

### 2.1. Heterologous Expression of HCN Channel Subunits

Human embryonic kidney cells (HEK293; #85120602) were obtained from ECACC/Sigma Aldrich (Taufkirchen, Germany) and grown in a low glucose containing medium (M10, MEM + Glutamax^TM^, 10% (*v*/*v*) fetal calf serum (FCS), 1% antibiotics/antimycotics, and 1% (*v*/*v*) non-essential amino acids (all from Gibco/Thermo Fisher Scientific, Darmstadt, Germany). The cells were propagated in 9 cm petri dishes at 37 °C, 5% CO_2_, and ~95% relative humidity. Twice a week, when the cells reached approximately 90% confluency, they were trypsinized and seeded at densities of 1–1.4 × 10^6^ cells onto new petri dishes. Transfections were performed with a modified calcium phosphate co-precipitation method in order to establish cell lines constitutively expressing individual HCN channel subunits (HCN1, 2, 4) [18]. The cell clones were selected in the presence of Geneticin (0.8–1 mg mL^−1^ in M10 medium). The functional expression of HCN channels and homogeneity of cell clones was examined electrophysiologically and by immunological staining, respectively.

### 2.2. Primary Hippocampal Neuron Cultures

Hippocampi were obtained from 1–3 days-old mice (C57BL/6 strain from an in-house animal facility) or a transgenic 129/Sv-based mouse line that does not express functional HCN1 channel proteins [19]. The animals of the transgenic strain were kindly provided by Dr. E. R. Kandel (Center for Neurobiology and Behavior, Columbia University, USA). Brains were dissected in ice-cold Hanks’ balanced salt solution (HBSS; Gibco/Thermo Fisher Scientific). Hippocampi were incubated in papain solution (DMEM (Gibco/Thermo Fisher Scientific), 25 U mL^−1^ papain, 1.6 mM L-cysteine, 1 mM CaCl_2_, 0.5 mM EDTA) at 37 °C for 20 min, and subsequently transferred to inactivating solution (2.5% (*w*/*v*) trypsin inhibitor, 2.5% (*w*/*v*) albumin in FCS solution consisting of DMEM, 100 U mL^−1^ penicillin, 100 μg mL^−1^ streptomycin, 10% (*v*/*v*) FCS; all from Gibco/Thermo Fisher Scientific), and 0.1% (*v*/*v*) MITO + serum extender (Corning/Thermo Fisher Scientific, Darmstadt, Germany) at 37 °C for 5 min. The cells were then triturated in FCS solution. Primary hippocampal neurons (PHNs) were counted and plated on coverslips in four-well plates (Ibidi, Martinsried, Germany) pre-coated with poly-D-lysine (0.2 mg mL^−1^ poly-D-lysine, 50 mM H_3_BO_3_, 25 mM Na_2_B_4_O_7_, pH 8.5). PHNs were plated with a density of 300 cells mm^−2^ and maintained in 500 µL NBA medium (Neurobasal A Medium (Gibco/Thermo Fisher Scientific), 100 U mL^−1^ penicillin, 100 μg mL^−1^ streptomycin, 2% (*v*/*v*) B27‑supplement (Invitrogen/Thermo Fisher Scientific, Darmstadt, Germany), and 1% (*v*/*v*) Glutamax (Gibco/Thermo Fisher Scientific)) at 37 °C, 5% CO_2_ and 95% relative humidity for 15 days. Medium was partially exchanged every 2–3 days. For transduction, recombinant Adeno-associated viral suspensions (rAAVs) were added with a multiplicity of infection of 2 × 10^4^ per neuron, 2–3 days after plating (days in vitro, d.i.v.).

### 2.3. Organotypic Hippocampal Slice Culture

Hippocampi from 2–3 days-old mice (C57BL/6 strain from an in-house animal facility) were dissected in ice-cold oxygenated dissection buffer (ACSF: 124 mM NaCl, 2.5 mM KCl, 1.25 mM NaH_2_PO_4_, 26 mM NaHCO_3_, 5 mM MgSO_4_, 0.5 mM CaCl_2_, 25 mM D-glucose). The hippocampi were placed on a PTFE membrane (Merck, Darmstadt, Germany) and cut into 300 µm coronal slices while using a custom-made tissue chopper. The slices were washed three times in pre‑warmed HBSS without Ca^2+^ and Mg^2+^ ions (Gibco/Thermo Fisher Scientific). Three slices were collected and positioned on Millicell cell culture inserts (30 mm, hydrophilic PTFE, 0.4 µm; Merck, Darmstadt, Germany) in six-well culture plates (Corning/Merck, Darmstadt, Germany) containing 1 mL of a well-established slice culture medium according to Stoppini et al. [20] with slight modifications (80% (*v*/*v*) MEM, 20% (*v*/*v*) heat inactivated horse serum (Gibco/Thermo Fisher Scientific) containing (in final concentrations) 20 mM HEPES, 14.5 mM NaCl, 2 mM MgSO_4_, 1 mM CaCl_2_, 1 mM L-glutamine, 80 µM ascorbic acid, 13 mM D-glucose, 0.033% (*v*/*v*) insulin, 50 U mL^−1^ penicillin, and 50 µg mL^−1^ streptomycin). The slice cultures were maintained at 5% CO_2_, 95% relative humidity and 37 °C for a maximum of 15 days. Medium was partially exchanged every 2–3 days. For transduction, a total of 1 × 10^8^ rAAVs were added on top of a slice at d.i.v. 1– 2 resulting in a similar MOI (~1 × 10^4^) as used for PHN transduction [21].

### 2.4. Immunocytochemistry

Table 1 and Table 2 list the primary and secondary antibodies used for immunocytochemistry (ICC), respectively. Cells or tissue slices were rinsed with PBS and then fixed in PFA (4% (*w*/*v*) paraformaldehyde in PBS) for 10 min at room temperature (RT). After several rinses with PBS, unspecific binding sites were blocked for 1 h at RT in blocking solution (CT: 0.5% (*v*/*v*) Triton X-100, 5% (*v*/*v*) ChemiBLOCKER (Merck, Darmstadt, Germany). Subsequently, the samples were incubated with primary antibodies (Table 1) in CT at 4 °C over night or at RT for 4 h, rinsed for several times with PBS, and then incubated with secondary antibodies (Table 2) in CT at RT for 1 h. Finally, the samples were washed with PBS, before mounting the coverslips containing cells or PTFE membranes containing slices in Aqua-Poly/Mount (Polysciences, Eppelheim, Germany) on microscopy slides. The fluorescent images were obtained with an inverted confocal microscope (TCS SP5II; Leica, Wetzlar, Germany).

### 2.5. Colocalization Analysis

HEK293 cell lines constitutively expressing individual HCN channel subunits (HCN1, 2, 4) were transfected with shRNA encoding plasmids using PEI transfection [22]. The cells were plated on coverslips in 24-well plates (Ibidi, Martinsried, Germany) that were pre-coated with poly-L-lysine (0.1 mg mL^−1^) with a density of 300 cells mm^−2^. Cultures were kept in a humidified CO_2_ incubator (5% CO_2_, 95% relative humidity) at 37 °C and maintained for two days before fixation. After immunocytochemistry and image acquisition, the images were analyzed using the ImageJ Coloc 2 analysis plugin [23].

### 2.6. Cloning of Constructs and Preparation of rAAVs

For targeting *hcn* genes by RNAi, several shRNA-encoding recombinant plasmids were purchased from Sigma Aldrich/Merck (Darmstadt, Germany). Individual fragments were cloned into the pENN‑CaMKIIeGFP vector provided by the University of Pennsylvania Vector Core (Philadelphia, PA, USA) containing the human U6 (hU6) promoter 5′ upstream to the shRNA-encoding fragment. For generating a CRISPRi vector, a custom designed backbone, including AAV serotype 2 wildtype (wt) inverted terminal repeats (ITRs), an HA-tag, and a KRAB element was purchased from Invitrogen/Thermo Fisher Scientific. The sgRNA scaffold, including the hU6 promoter and a dSaCas9-encoding construct, were a gift from Feng Zhang (Addgene (Watertown, MA, USA) plasmid #61591 for the sgRNA scaffold and SaCas9; plasmid #61594 for dSaCas9) [24]. *Hcn*-gene specific sgRNA sequences were designed using E-CRISP, a fast CRISPR target-site identification online tool [25]. Complementary single-stranded oligonucleotides were purchased from Eurofins. Complementary pairs of oligonucleotides were incubated at 95 °C for 5 min in 50 mM NaCl, 1 mM EDTA, 10 mM Tris/HCl (pH 7.5), and annealed while cooling the samples to RT. Double-stranded fragments were cloned into the sgRNA scaffold of the vector plasmid. Recombinant Adeno-associated viral (rAAV) particles were prepared in-house, as previously described by [26]. In brief, the HEK293 cells were triple-transfected (ATCC; #CRL-1573) using the modified calcium phosphate coprecipitation method [18]. The HEK293 cells were cultivated in DH10 medium (DMEM + Glutamax^TM^, 10% (*v*/*v*) FBS, 1% (*v*/*v*) antibiotics/antimycotics (all from Gibco/Thermo Fisher Scientific)) at 37 °C, 5% CO_2_, and 95% relative humidity. After 24 h, the cells were triple transfected with AAV2 wt ITR-flanked vectors containing the transgenic viral genome and the helper plasmids pXX6-80 and pRC2 or pRC9 providing the proteins for DNA replication and capsid assembly of rAAVs. 24 h after transfection the medium was exchanged for hunger medium (DH10, 2% (*v*/*v*) FBS, and 1% (*v*/*v*) antibiotics/antimycotics (all from Gibco/Thermo Fisher Scientific)). 72 h after transfection, the cells were harvested in PBS‑M/K (130 mM NaCl, 2.5 mM KCl, 1 mM MgCl_2_, 70 mM Na_2_HPO_4_, 30 mM NaH_2_PO_4_, pH 7.4) and centrifuged (200 g, 4 °C, 5 min). Cell pellets were re-suspended in lysis buffer (150 mM NaCl, 50 mM Tris/HCl, pH 8.5) and cells were lyzed by five freeze/thaw-cycles. Free nucleic acids were digested with benzonase (50 U/mL; Merck Millipore, Darmstadt, Germany) for 30 min at 37 °C. After a centrifugation step (5000 g, 4 °C, 30 min), the rAAV suspension was sub‑layered with iodixanol solutions (15%, 25%, 40%, and 60% iodixanol; Sigma‑Aldrich/Merck) and centrifuged (264,000 g, 4 °C, 2 h). The viral particles were collected in the 40% iodixanol phase, sterile filtered (0.2 µm pore size), and further purified using Amicon Ultra Centrifugal Filters (Ultracel‑100k, 15 mL; Merck Millipore). For the determination of genomic titers, viral genomes were isolated using the DNeasy Blood & Tissue Kit (Qiagen, Hilden, Germany) and quantitative PCR was performed using primers framing either a segment of the eGFP‑encoding sequence or the dSaCas9-encoding sequence (Table 3).

### 2.7. Quantification of Gene Expression by Real-Time qPCR

Total RNA was isolated from PHNs or organotypic hippocampal slice cultures (OHSCs) after 14–15 d.i.v. using the RNeasy Mini Kit (Qiagen) according to the supplier’s protocol. First-strand cDNA synthesis was performed according to the methods that were described in [27]. In brief, Oligo-dT primers (Qiagen) and Moloney Murine Leukemia Virus reverse transcriptase (M-MLV-RT, Life Technologies/Thermo Fisher Scientific) were used according to the supplier’s protocol. Thermocycling was performed in a LightCycler 1.5 (Roche, Mannheim, Germany) using the QuantiTect SYBR Green PCR Kit (Qiagen). qPCR reactions were performed on 2 µL aliquots of first‑strand cDNA samples in a total volume of 20 µL. The results were analyzed using the C_t_ method. Gene-specific primers (Table 3) were designed in silico and synthesized by Eurofins (Ebersberg, Germany). The specificity of primers was confirmed via BLAST analysis. For normalization, *gapdh* was used as a reference gene. The *gapdh* primers were designed to bind in exons that are separated by an intron of 134 bp to check for genomic impurities.

### 2.8. Validation of Knock-Down Efficiencies for shRNAs/sgRNAs

ShRNA-encoding constructs were transfected by calcium phosphate co-precipitation into HEK293 cell lines constitutively expressing individual HCN channel subunits (HCN1, 2, 4). For each target gene, up to five different shRNA sequences (see Table 4 and Supplementary Table S1) were independently tested. The total RNA was isolated from HEK293 cells using the AllPrep DNA/RNA/Protein Mini Kit (Qiagen) according to the supplier’s protocol. SgRNA-encoding constructs were packed into viral particles of serotype 2 and PHNs were transduced with a MOI of 2 × 10^4^. For each target gene, up to four different sgRNA sequences were tested independently. Total RNA was isolated from PHNs using the RNeasy Mini Kit (Qiagen), according to the supplier´s protocol. Those constructs that induced a robust and reproducible knock-down were chosen for further analysis.

### 2.9. Whole-Cell Patch-Clamp Analysis

Whole-cell patch-clamp recordings were performed at RT following the methods that were described by Hamill et al. [29]. Electrodes with tip resistances between 2.5 and 4 MΩ were fashioned from borosilicate glass with an inner diameter of 0.86 mm and an outer diameter of 1.5 mm (Harvard Apparatus, Holliston, MA, USA) with a temperature-controlled pipette puller (P1000, Sutter Instrument, Novato, CA, USA). For HEK293 cells and PHNs, the pipettes were filled with intracellular saline solution containing 10 mM KCl, 10 mM NaCl, 120 mM KGluconate, 10 mM EGTA, 10 mM HEPES, 4 mM MgATP, and 0.3 mM NaGTP, adjusted to pH 7.3 with KOH and an osmolality of ~310 mOsm/L. During the experiments, the cells were constantly superfused with extracellular saline solution containing 150 mM NaCl, 4 mM KCl, 2 mM CaCl_2_, 2 mM MgCl_2_, and 10 mM HEPES, adjusted to pH 7.4 with NaOH and adjusted to 330 mOsm/L with glucose. Whole-cell voltage-clamp and current-clamp recordings were performed while using an EPC10 patch-clamp amplifier (HEKA-Elektronik, Lambrecht, Germany) that was controlled by the program Patch Master (version 2.5; HEKA-Elektronik). Electrophysiological data were sampled at 20 kHz and low pass filtered at 2.9 kHz with a four-pole Bessel-filter. Offset potentials, electrode capacity, and membrane capacity were manually compensated. The liquid junction potential between intracellular and extracellular solutions was calculated and also compensated by adjusting the offset potential. Series resistance was compensated between 60 and 80% with a time constant (τ) of 100 μs.

### 2.10. Data Analysis

Electrophysiological data were analyzed using FitMaster (version 2; HEKA-Elektronik) or Igor Pro (version 6; Wavemetrics, Lake Oswego, OR, USA). The data are represented as mean ± s.d. (standard deviation) or as box and whisker plots. The two-tailed unpaired Student’s t test was applied for the calculation of *p* values using GraphPad Prism (version 5; Graphpad Software Inc., La Jolla, CA, USA). A *p* value of <0.05 was considered to be significant.

## 3. Results

### 3.1. Generation of AAV-Based Gene-Interference Tools

**Figure 1:**
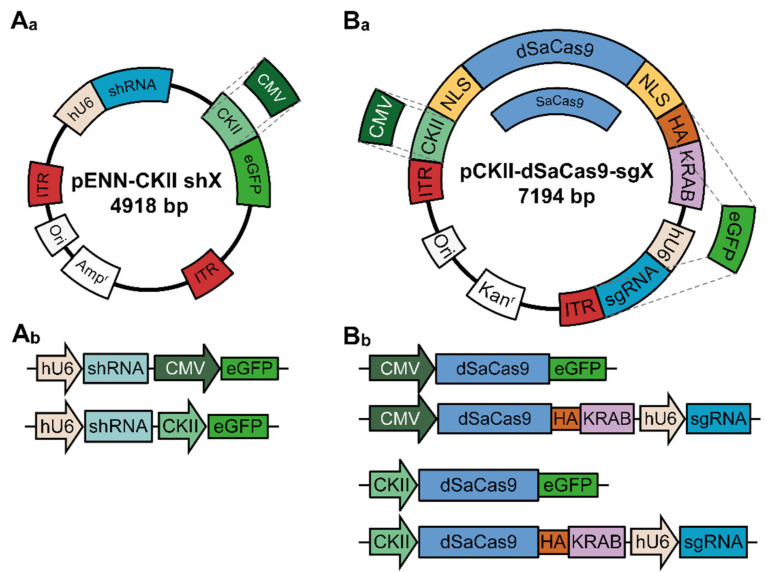
Construction of shRNA-encoding and all-in-one dSaCas9-encoding vectors for Adeno-associated viruses (AAV)‑mediated RNA interference (RNAi) and CRISPRi. (**A_a_**) Schematic representation of the shRNA expression plasmid. (**A_b_**) Schematic representation of shRNA-encoding constructs used in this study. Expression of the eGFP reporter could be driven by the CMV- or CKII promoter. (**B_a_**) Schematic representation of the dSaCas9 and sgRNA expression plasmid. (**B_b_**) Schematic representation of sgRNA and dSaCas9-encoding constructs used in this study. Expression of the eGFP reporter could be driven by the CMV- or CKII promoter. For control experiments, variants with dSaCas9 fused to eGFP were generated. The modulatory design of the vector also allows to exchange dSaCas9 to SaCas9 for conventional CRISPR experiments. Abbreviations: ITR, inverted terminal repeat; hU6, human U6 promoter; shRNA, short-hairpin RNA; CKII, CaM kinase II promoter; CMV, cytomegalovirus promoter; eGFP, enhanced green fluorescent protein; Ori, origin of replication; Amp^r^, ampicillin resistance cassette; NLS, nuclear localization site; dSaCas9, nuclease deficient *Staphylococcus aureus* Cas9; HA, human influenza hemagglutinin tag; KRAB, Krüppel-associated box; sgRNA, short-guidance RNA; Kan^r^, kanamycin resistance cassette.

In addition to transgenic approaches, gene expression can be manipulated alternatively by the cell-autonomous defense mechanism of RNA inhibition (RNAi) [30]. By forming homologous RNA double strands, the degradation of the targeted mRNA is initiated. Consequently, the protein level decreases due to an impaired *de novo* protein biosynthesis. The core component necessary for RNAi is a short hairpin RNA (shRNA) that is expressed, e.g., under the control of constitutively active human U6 (hU6) promoter. In this study, different shRNA molecules were designed complementary to the target HCN channel-encoding mRNA molecules. For the identification of transduced cells, an eGFP-reporter expressed either under the control of the neuron-specific CaM kinase II (CKII) promoter or the CMV promoter was used (Figure 1(A_a_,A_b_)). In all experiments that were conducted with neurons, constructs were delivered by recombinant Adeno-associated viruses (rAAVs).

As an alternative strategy, a modified version of the “Clustered Regularly Interspaced Short Palindromic Repeats” (CRISPR) technique was applied and compared with the RNAi strategy. The CRISPR technique typically leads to a gene knock-out initiated by enzymatically active Cas9 proteins, cutting the genomic DNA. The DNA double strand break triggers cellular repair mechanisms, which eventually cause a gain or loss of nucleotides that ultimately disturb a genes’ open reading frame [31,32,33,34,35]. In contrast, the CRISPR interference (CRISPRi) strategy blocks RNA synthesis by the occupation of the target gene’s promoter and thereby impairs protein *de novo* biosynthesis [36,37,38]. The main component of CRISPRi is a nuclease-deficient version of Cas9 (dSaCas9), which is fused to the Krüppel-associated box (KRAB) repression domain, resulting in a transcriptional interference protein complex (dSaCas9-KRAB; Figure 1(B_a_,B_b_)) [37,39]. The expression of this fusion protein was driven either by the CKII- or the CMV promoter. Because of the flanking nuclear localization sites, the dSaCas9-KRAB complex translocates into the nucleus, where, assisted by short guidance RNAs (sgRNAs), it binds to the transcriptional start site (TSS) of the target gene and interferes with the transcription machinery. Similar to shRNAs, the sgRNAs were expressed under control of the hU6 promoter. For the identification of transduced cells, an HA-tag was fused to the dSaCas9 protein. We generated an AAV-vector backbone in which the sgRNA expression scaffold, the HA-tag, and the KRAB motif can be exchanged for an eGFP-encoding cassette that is directly fused to the Cas9-encoding element in order to achieve more flexibility and allow direct proof of Cas9 expression (Figure 1(B_a_,B_b_)).

### 3.2. Functional Testing of Knock-Down Constructs

**Figure 2:**
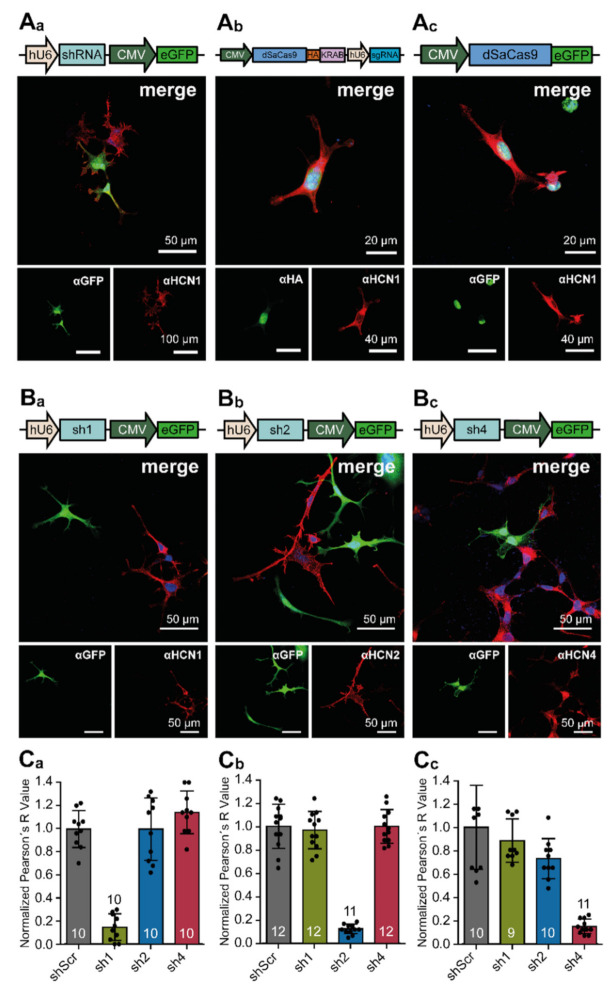
Expression of RNAi- and Clustered Regularly Interspaced Short Palindromic Repeats interference (CRISPRi)-mediating constructs in HEK293 cells. (**A_a_**–**A_c_**) Representative immunofluorescence images showing the expression of the (**A_a_**) eGFP reporter of the RNAi construct, (**A_b_**) dSaCas9 protein including the KRAB domain and the sgRNA expression scaffold, and (**A_c_**) eGFP-tagged dSaCas9 in HEK293 cells constitutively expressing the hyperpolarization activated and cyclic nucleotide-gated (HCN) channel isoform 1 (HCN1). The eGFP, HA-tag, and HCN1 proteins were immunostained with specific antibodies. (**B_a_**–**B_c_**) Representative immunofluorescence images showing the expression of (**B_a_**) the eGFP reporter of sh1-expressing, (**B_b_**) sh2-expressing, or (**B_c_**) sh4-expressing variants in HEK293 cells constitutively expressing HCN channel isoforms 1, 2, or 4. Staining was performed with specific anti-eGFP, anti-HA-tag, and anti-HCN antibodies combined with fluorescently labeled secondary antibodies (green and red). Nuclei were labeled with TOPRO (blue). (**C_a_**–**C_c_**) Colocalization analysis by comparison of Pearson’s R values for HEK293 cells (**C_a_**) constitutively expressing HCN1 channels and different shRNAs (shScr, sh1, sh2 and sh4), (**C_b_**) constitutively expressing HCN2 channels and different shRNAs (shScr, sh1, sh2 and sh4), and (**C_c_**) constitutively expressing HCN4 channels and different shRNAs (shScr, sh1, sh2 and sh4). The data were obtained from indicated numbers of fluorescent images from at least five independent transfections. Pearson’s R values were normalized to shScr controls and results are depicted as mean ± standard deviation. Schematic of AAV-delivered constructs are displayed above the merged immunofluorescence images.

In order to check for the functionality of the different knock-down constructs, we transfected HEK293 cells and examined the expression and localization of the eGFP reporter as well as HA-tagged dSaCas9. Figure 2(A_a_) shows the expression of eGFP under control of the CMV promoter. The distribution of eGFP was not restricted to specific cellular compartments. Figure 2(A_a_–A_c_) show the expression of the dSaCas9 either fused to an HA-tag (2A_b_) or to an eGFP (2A_c_). The protein was preferentially located in the nucleus, due to nuclear localization sites (NLS) flanking the dSaCas9 cassette (see Figure 1(B_a_,B_b_)).

shRNA-encoding constructs targeting individual HCN channel isoforms were transfected in HEK293 cells constitutively expressing homomeric HCN channels 1, 2, or 4 in order to validate the specificity and efficacy of the RNAi-mediated knock-down on the protein level. Two days after transfection, the cells were fixed and stained with specific antibodies (Figure 2(B_a_–B_c_)). Capturing confocal images of HCN channel fluorescence and eGFP fluorescence allowed for calculating Pearson R values for the colocalization of eGFP and HCN channel signals. The immunofluorescence signal of the HCN channel will decrease in those cells in which HCN channel expression is downregulated by shRNAs, whereas the eGFP-reporter signal remains constant. Consequently, the Pearson R value decreases as a measure for colocalization of both signals. Normalized colocalization analyses are shown in Figure 2(C_a–_C_c_). Only shRNA1 (sh1), which is complementary to *hcn1* mRNA downregulated HCN1 channel protein expression (Figure 2(C_a_)), whereas the control shRNA (shScr) and shRNAs complementary to *hcn2* and *hcn4* mRNAs did not change the expression level of HCN1 (shScr: 1.0 ± 0.159; sh1: 0.149 ± 0.115). Similarly, only shRNA2 (sh2), which is complementary to *hcn2* mRNA (shScr: 1.0 ± 0.189; sh2: 0.122 ± 0.042) (Figure 2(C_b_)), and shRNA4 (sh4), which is complementary to *hcn4* mRNA (shScr: 1.0 ± 0.361; sh4: 0.152 ± 0.062) (Figure 2(C_c_)) induced the specific downregulation of their target transcripts and a reduction in channel protein expression.

**Figure 3:**
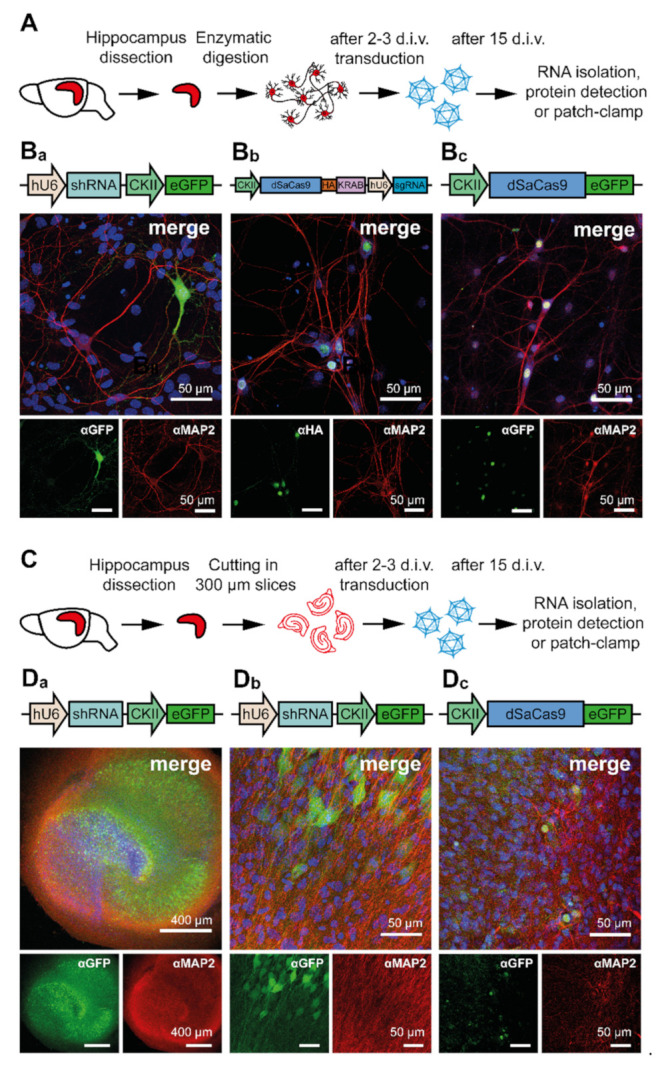
AAV-mediated expression of different constructs in primary hippocampal neurons (PHNs) and organotypic hippocampal slice cultures (OHSCs). (**A**) Schematic representation of the preparation and transduction procedure of primary hippocampal neurons (PHNs). (**B_a_**–**B_c_**) Representative immunofluorescence images of rAAV9-transduced PHNs expressing the (**B_a_**) eGFP reporter, (**B_b_**) HA-tagged dSaCas9, and (**B_c_**) eGFP-tagged dSaCas9. (**C**) Schematic representation of the preparation and transduction procedure for OHSCs. For details see Material and Methods. (**D_a_**–**D_c_**) Representative immunofluorescence images showing rAAV9-transduced OHSCs expressing the eGFP reporter (**D_a_**,**D_b_**), or eGFP-tagged dSaCas9 (**D_c_**). The eGFP, HA-tag, and the neuron-specific microtubule-associated protein 2 (MAP2) protein were immunostained with specific anti-GFP, anti-HA, and anti-MAP2 antibodies combined with fluorescently labeled secondary antibodies (eGFP and HA-tag, green; MAP2, red). Nuclei were labeled with TOPRO (blue). Schematic of AAV-delivered constructs are displayed above the merged immunofluorescence images.

From all of the constructs tested (see Table 3 and Supplementary Table S1), only those shRNAs reliably demonstrating the highest specificity and efficacy were selected to generate rAAVs. Primary cultures of hippocampal neurons that were prepared from C57BL/6J mice (Figure 3A) were transduced with these rAAVs. The vectors either encoded shRNA and eGFP (Figure 3(B_a_)), dSaCas9-HA (Figure 3(B_b_)), or dSaCas9-eGFP (Figure 3(B_c_)). Twelve days after transduction, neurons were fixed and the expression of reporter proteins was studied by immunocytochemistry. Similar to the expression pattern in HEK293 cells, in neurons expressing shRNAs, eGFP expression was not restricted to a specific cellular compartment. The dSaCas9 fused either to the HA-tag or to eGFP was located in the nucleus.

### 3.3. AAV-Mediated Knock-Down of hcn Gene Expression in Neurons

**Figure 4:**
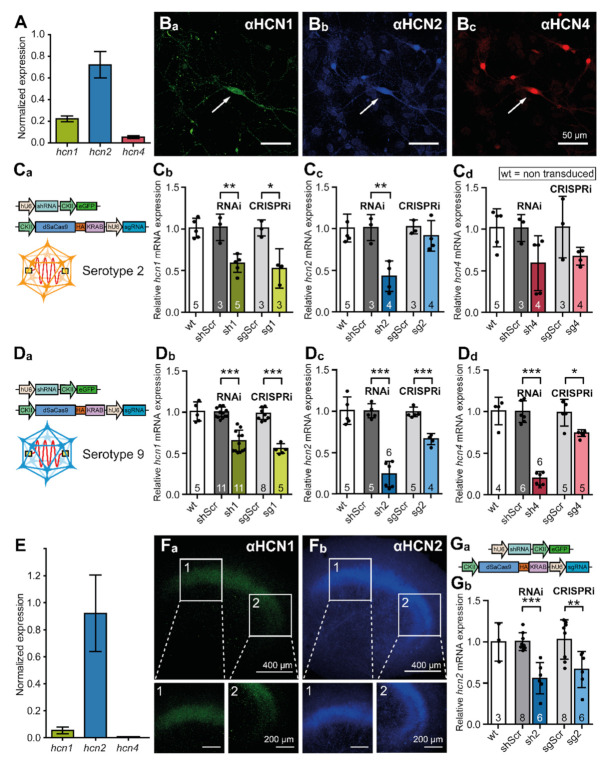
RNAi and CRISPRi reduce the amount of HCN channel transcripts in PHNs and OHSCs. (**A**) qRT-PCR analysis of transcript expression levels for HCN isoforms 1, 2, and 4 in primary hippocampal neurons (PHNs). Transcript expression levels were normalized to *gapdh.* Values shown are calculated to 1 as the sum of all *hcn* transcripts. cDNA was prepared from 5 coverslips with PHNs from at least three different animals. (**B_a_**–**B_c_**) Representative immunofluorescence images showing expression of HCN-channel isoforms 1 (green), 2 (blue) and 4 (red). Isoforms were stained using specific antibodies combined with fluorescently labeled secondary antibodies. (**C_a_**) Schematic representation of constructs delivered by rAAV2 to PHNs. (**C_b_**–**C_d_**) qRT-PCR analysis of *hcn1*, *hcn2*, and *hcn4* mRNA levels in hippocampal neurons after transduction with shRNA or sgRNA/dSaCas9 expressing rAAVs. (**D_a_**) Schematic representation of constructs delivered by rAAV9 to PHNs. (**D_b_**–**D_d_**) qRT-PCR analysis of *hcn1*, *hcn2*, and *hcn4* mRNA levels in hippocampal neurons after transduction with shRNA or sgRNA/dSaCas9 expressing rAAVs. (**E**) qRT-PCR analysis of transcript expression levels for HCN isoforms 1, 2, and 4 in organotypic hippocampal slice cultures (OHSCs). The transcript expression levels were normalized to *gapdh.* Values shown are calculated to 1 as the sum of all *hcn* transcripts. cDNA was prepared from five culture inserts, each containing three individual slices. In total, slices were obtained from three different animals. (**F_a_**,**F_b_**) Representative immunofluorescence images showing the expression of HCN-channel isoforms 1 (green) and 2 (blue). The isoforms were stained using specific antibodies. Enlargements show HCN-isoform expression in hippocampal CA1 (1) and CA3 (2) subfields. (**G_a_**,**G_b_**) qRT-PCR knock-down analysis of *hcn2* mRNA levels in organotypic slices after transduction with shRNA or sgRNA/dSaCas9 expressing rAAV9. The data were obtained from indicated numbers of culture inserts, each containing three individual slices. In total, slices were obtained from at least three different animals. The results are depicted as mean ± standard deviation. Statistical significance was assessed using the unpaired two-tailed Student’s *t* test, * *p* < 0.05, ** *p* < 0.01, *** *p* < 0.001.

Cultured PHNs express at least three HCN channel isoforms that can be quantified by qRT-PCR (Figure 4A). The analyses showed that *hcn2* transcripts were most abundant (72 ± 12.3%), followed by *hcn1* and *hcn4* transcripts (*hcn1*: 22 ± 2.6%; *hcn4*: 6 ± 0.9%). Even individual neurons expressed all three isoforms, as illustrated by immunocytochemistry (Figure 4(B_a_–B_c_)). Therefore, we aimed to downregulate each of the channel isoforms specifically and independently by RNAi and CRISPRi in order to determine the strategy best suited for *hcn* gene knock-down in postmitotic cells. For CRISPRi-mediated knock-down, sgRNAs were designed to target regions between −50 and + 300 bp relative to the predicted TSS. The knock-down efficacies of these sgRNAs were compared to a scrambled sgRNA (sgScr), which was designed not to bind to any endogenous promoter. In order to evaluate the CRISPRi efficacy, we also determined the effects of RNAi using the shRNAs that were previously validated in the colocalization experiments. For each construct, rAAVs representing serotype 2 and 9 were generated. While rAAV2 is considered to transduce a broad range of cell types and tissues, rAAV9 is more efficient in transducing neurons [40]. In neurons that were transduced with rAAV2 virions, each of the previously identified shRNAs reduced the mRNA levels of the target gene in comparison to untreated wildtype and shScr-treated controls (Figure 4(C_a_–C_d_)). Sh1 reduced the expression of *hcn1* transcripts to 58.3 ± 11.2% of the shScr control level. Similarly, sh2 reduced the expression of *hcn2* transcripts to 42.3 ± 18.3% of the shScr control level and sh4 reduced expression of *hcn4* transcripts to 60.1 ± 32.9% of the shScr control level. Notably, only one sg construct (sg1) caused a robust reduction of *hcn1* mRNA to a level of 51.8 ± 23.6% of the sgScr control (Figure 4(C_a_–C_d_)). Using rAAV9 virions for transduction (Figure 4(D_a_–D_d_)), the knock-down efficiencies were: sh1: 65.2 ± 13% of the shScr control level; sh2: 24 ± 15.5% of the shScr control level; and, sh4 25.2 ± 8.1% of the shScr control level). The knock-down efficiencies of sg1, sg2, and sg4, were markedly improved when delivered by rAAV9 virions (sg1: 71.1 ± 21.5% of the sgScr control level; sg2: 66.3 ± 6.6% of the sgScr control level; sg4 83.9 ± 4.1% of the sgScr control level). However, when compared to RNAi, CRISPRi inhibited *hcn1*, *2*, and *4* gene expression moderately.

While primary hippocampal neurons are perfectly suited to study a neurons’ physiology on the single cell level, organotypic hippocampal slice cultures (OHSCs) are widely used to study network properties. Therefore, we aimed to examine the effects of HCN channel knock-down in OHSCs that were prepared from C57BL/6J mice (Figure 3C). After two weeks of cultivation and 12–13 days after transduction with rAAV9 expressing shRNA and eGFP (Figure 3(D_a_,D_b_)) or dSaCas9-eGFP (Figure 3(D_c_)), the samples were fixed, and the expression of reporter proteins was examined immunohistochemically. In agreement with the previous experiments performed on PHNs, the transduction of OHSCs with rAAV9 virions encoding shRNAs yielded a high number of transduced neurons. Furthermore, rAAVs inducing RNAi and rAAVs causing CRISPRi both reproduced the localization pattern of reporter proteins previously observed in PHNs. The eGFP reporter that was encoded by shRNA containing constructs was homogenously distributed in the cell, whereas eGFP-dSaCas9 was restricted to the nucleus (Figure 3(D_a_–D_d_)).

OHSCs express *hcn1*, *hcn2*, and *hcn4* transcripts, as well as HCN1, HCN2, and HCN4 channel proteins (Figure 4(E,F_a_,F_b_)). qRT-PCR and immunohistochemistry both indicated that HCN2 is the most abundant channel isoform in OHSCs (qRT-PCR quantification: *hcn1* 5.3 ± 2.5%; *hcn2* 92.1 ± 28.4%; *hcn4* 5.5 ± 1.3%). Thus, we decided to exemplarily manipulate HCN2 channel expression in OHSCs. sh2 and sg2 both reduced *hcn2* mRNA levels when compared to untreated wildtype and shScr- or sgScr-treated controls (sh2: 52.1 ± 18.9%; sg2: 62.7 ± 21.9%) (Figure 4(G_a_,G_b_)). Therefore, we conclude that both of the techniques are suitable for manipulating the expression levels of *hcn* transcripts in a variety of culture systems.

### 3.4. Electrophysiological Characterization of HCN4 Channel Knock-Down

Because RNAi efficiently reduced HCN channel transcript levels in HEK293 cells, PHNs, and OHSCs, we decided to evaluate the effects of *hcn4* gene knock-down on both basic neuronal parameters and I_h_-current characteristics while using standard electrophysiological patch-clamp experiments.

Here, we examined PHNs with reduced levels of the HCN4 channel isoform. The results showed that a reduction of HCN4 expression did not influence basic cellular parameters of PHNs (Supplementary Figure S4). There was neither a change in resting membrane potential (shScr: −68.35 ± 3.9 mV; sh4 −68.8 ± 2.16 mV), input resistance (shScr: 382.7 ± 104.2 MΩ; sh4: 372.7 ± 130.4 MΩ) (Supplementary Figure S4A,B), nor in HCN channel-specific parameters, like I_h_-current amplitude (shScr 146.6 ± 117.4 pA; sh4: 174.7 ± 109.7 pA) or in Sag potential amplitude (shScr: −21.13 ± 7.63 mV; sh4: −24.28 ± 7.86 mV) (Supplementary Figure S4C,D).

**Figure 5:**
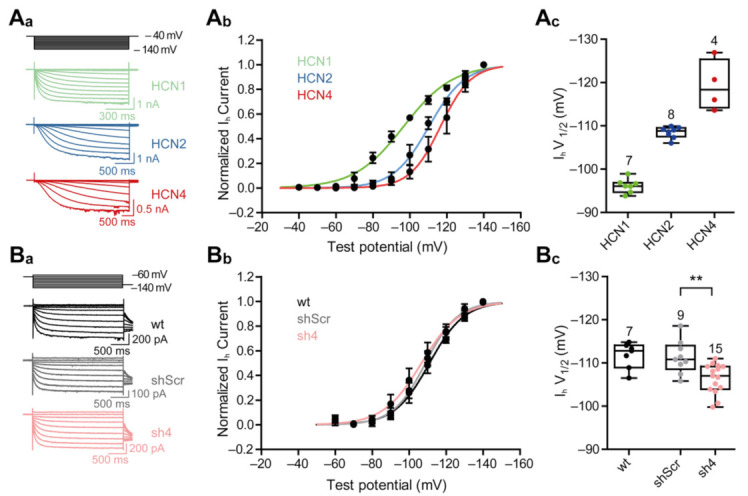
Knock-down of HCN-channel subunit four shifts half-maximal activation voltage of I_h_. (**A_a_**) Representative current traces of whole-cell patch-clamp recordings from −40 to −140 mV (Δ−10 mV) of HEK293 cells constitutively expressing HCN-channel subunit 1, 2, or 4. (**A_b_**) Activation curves of HCN-channel subunits 1, 2 and 4 measured from steady-state currents. The continuous lines represent fits to the Boltzmann function of the data. (**A_c_**) Half-maximal activation voltages of the different HCN-channel subunits, converted from the Boltzmann functions of whole-cell currents. (**B_a_**) Representative current traces of whole-cell patch-clamp recordings from −60 to −140 mV (Δ−10 mV) of wildtype (wt, non-transduced) primary hippocampal neurons (PHNs) or rAAV9-transduced eGFP-positive PHNs, either expressing sh4 or shScr. (**B_b_**) Activation curves of wt and transduced PHNs. The continuous lines represent fits to the Boltzmann functions of the data. (**B_c_**) Half-maximal activation voltages wt and sh4- or shScr-transduced PHNs, derived from the Boltzmann functions of whole-cell currents. The results are depicted as boxplots. Statistical significance was assessed using the unpaired two-tailed Student’s *t* test, ** *p* < 0.01.

To obtain detailed insight into HCN4 subunit-specific functions, we measured I_h_-currents at different membrane potentials and constructed current–voltage relationships, from both HEK293 cells expressing homomeric HCN channels and PHNs expressing heteromeric HCN channels (Figure 5(A_a_–A_c_,B_a_–B_c_)). Half-maximal activation voltages recorded from HEK293 cells confirmed the differences in activation potentials of homomeric HCN channel currents [9]. While homomeric HCN1 channels activate at more positive membrane potentials, HCN2 and HCN4 homomeric channels activate at more negative membrane potentials (HCN1: −96.14 ± 1.63 mV; HCN2: −108.5 ± 1.3 mV; HCN4: −119.3 ± 5.86 mV), thus indicating an activation sequence of HCN1 > HCN2 > HCN4 from more depolarized to very hyperpolarized potentials (Figure 5(A_a_–A_c_)). In PHNs, the knock-down of HCN4 leads to a shift in half-maximal activation voltage of about 5 mV from hyperpolarized to more depolarized potentials as compared to shScr-treated control neurons (shScr: −111.3 ± 3.84 mV; sh4: −106.4 ± 3.42 mV) (Figure 5(B_a_–B_c_)).

**Figure 6:**
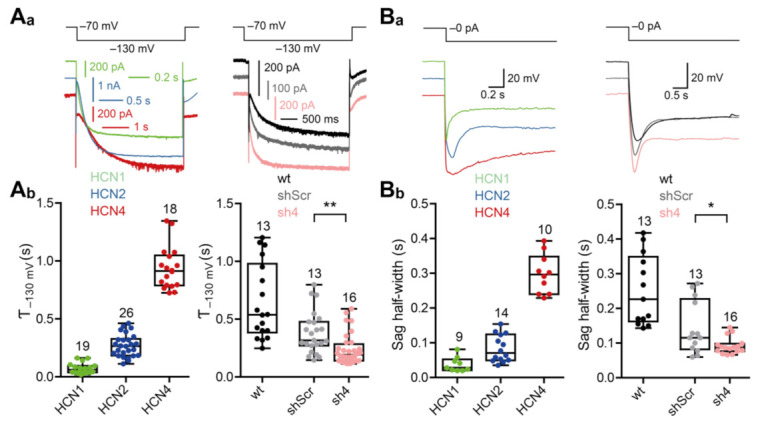
Knock-down of HCN-channel subunit 4 speeds up the inward rectification (sag) and I_h_. (**A_a_**) Representative current traces of whole-cell patch-clamp recordings from −130 mV current pulses of HEK293 cell lines (left) and wildtype (wt) or sh4-/shScr-transduced primary hippocampal neurons (PHNs; right). (**A_b_**) Time constants of monoexponential fits from the current responses of the HEK293 cell-lines (left) and wt or sh4-/shScr-transduced PHNs (right). (**B_a_**) Representative voltage traces of whole-cell patch-clamp recordings from current pulses which hyperpolarize the membrane potential to −130 mV of HEK293 cells stably expressing HCN-channel subunit 1, 2, or 4 (left) and wt or rAAV9-transduced eGFP-positive PHNs (right). (**B_b_**) Sag half-width of HEK293 cell-lines (left) and wildtype or transduced PHNs (right). Results are depicted as boxplots. Statistical significance was assessed using the unpaired two-tailed Student’s *t* test, * *p* < 0.05, ** *p* < 0.01.

We measured the activation time constants (τ) of homomeric HCN channels in HEK293 cells and of HCN channels endogenously expressed in PHNs in order to analyze activation kinetics of HCN channel-mediated I_h_-currents (Figure 6(A_a_,A_b_); see also Supplementary Figures S5 and S6). Similar to the differences in activation potentials, homomeric HCN channel currents differed in their activation kinetics, as illustrated by differences in τ values derived from I_h_-currents measured at −130 mV. While homomeric HCN1 channels activated with a time constant of 71.6 ± 43.9 ms, homomeric HCN2 channels activated with a time constant of 269.7 ± 97 ms and homomeric HCN4 channels activated even slower with a time constant of 934.4 ± 183.2 ms. The differences in the activation kinetics were also apparent in the half-width of Sag-potentials. While half-widths of Sag potentials from homomeric HCN1 channels were 37.9 ± 21 ms, half-widths of Sag potentials from homomeric HCN2 and HCN4 channels were 83.29 ± 38.9 ms and 298.6 ± 56.47 ms, respectively. The knock-down of HCN4 in PHNs increased the speed of activation of endogenous HCN channels when compared to shScr-treated neurons. This increase in kinetics resulted in faster activation time constants (shScr: 382 ± 181.1 ms; sh4: 238.1 ± 131.6 ms) (Figure 6(A_a_,A_b_)) and also in smaller Sag potential half-width values (shScr: 143.2 ± 76.27 ms; sh4: 92.06 ± 23.82 ms) (Figure 6(B_a_,B_b_)). Thus, the knock-down of HCN4 spares an I_h_ current that has inherent characteristics of heterotetrameric currents that are mainly composed of HCN1 and HCN2 channel subunits.

## 4. Discussion

For studying the signal transduction and communication pathways in biological systems, there is a need for applying methodologies that allow less invasive approaches to assess the function of individual proteins in single cells and on the network level in tissue or in living organisms. In comparison to transgenic approaches that are time consuming and bear the risk of unexpected developmental defects, methods to impair transcript levels of a gene of interest, i.e., gene knock-down strategies, hold great promise in overcoming the limitations of genome changing or editing strategies.

Here, we examined two independent experimental approaches, i.e., RNAi and CRISPRi, in order to reduce the expression of HCN channel-encoding genes at the mRNA level without altering the gene’s nucleotide sequence. The main finding of our study was that both procedures reduced *hcn* transcript levels in transgenic cell-lines, primary hippocampal neurons (PHNs), and organotypic hippocampal slice cultures (OHSCs) for each of the three targeted HCN isoforms. We used rAAVs as cargo vehicles to overcome constraints of construct delivery to neurons. Because rAAV serotypes differ in their transduction efficacy for certain cell types or tissues, we used rAAV2 virions that are known to transduce a broader spectrum of cell types, and rAAV9 virions that are known to be particularly suited for neuronal and glial cell transduction, respectively [41,42]. Furthermore, the knock-down of HCN4 channel subunits in PHNs unraveled unique insights into single cell I_h_-current related electrophysiological properties.

The main goal of this study was to examine the specificity and efficacy of two independent strategies to uncover a suitable system for knock-down applications of *hcn* gene expression in forthcoming studies. Initially, an all-in-one CRISPRi system was developed and tested in recombinant cell-lines and PHNs. In contrast to conventional CRISPR gene-editing tools [31,32,33,34,35], CRISPRi is based on a mutation in the Cas9 gene, leading to a loss of nuclease activity of the enzyme (dSaCas9) [17,36,37,38]. The dSaCas9-encoding cDNA was fused to the Krüppel-associated box (KRAB) repression domain to generate a protein complex that is able to interfere with gene transcription [27,39]. The constructs were delivered via rAAVs, which are known to be non-immunogenic and they have been approved for therapeutic applications [43,44]. However, the maximal cargo size of AAV genomes is ~4.5 kb [45,46]. Incorporating the native *Streptococcus pyogenes* Cas9 (SpCas9) gene (4.2 kb) [31] and a sgRNA-encoding cassette into the vector backbone exceeded the size limits of the virion’s capsid. In order to overcome this limitation, the SpCas9-encoding gene was substituted for an orthologous gene, previously identified in *Staphylococcus aureus* (SaCas9). The SaCas9-encoding cDNA has a size of 3.2 kb and the inactive version of the enzyme has been generated (dSaCas9) [24]. Thus, the all-in-one cloning vector (see Figure 1) contained the enzymatically inactive dSaCas9 fused to the Krüppel-associated box repression domain (dSaCas9-KRAB) and it was flanked by nuclear localization sites facilitating nuclear translocation of the fusion protein. The expression of the dSaCas9-KRAB protein was driven by exchangeable RNA polymerase II promoters, with the CMV-promoter being active in a wide range of cell types, whereas the CKII-promoter is preferentially active in neurons. sgRNA-encoding elements controlled by an RNA polymerase III promoter (human U6-promoter) were integrated to achieve specific binding of dSaCas9-KRAB to the promoter region of a gene of interest. The modular vector design also allowed for expressing fluorescent reporters, like eGFP, to visualize the transduction success. Finally, enzymatically active SaCas9 can be substituted for dSaCas9-KRAB to perform conventional CRISPR gene-editing experiments. The versatility and functionality of the vector was confirmed in independent experiments showing that the dSaCas9-KRAB protein was restricted to the nucleus of transfected HEK293 cells (Figure 2(A_a_–A_c_)) and transduced PHNs or OHSCs (Figure 3(B_a_–B_c_/3D_a_–D_c_)).

Because the promoters of murine HCN channel genes have not been experimentally determined, we used the Eukaryotic Promoter Database (EPD) [25] to predict the TSS of the mouse *hcn1, 2*, and *4* genes. For each of these genes, we designed at least three independent sgRNAs (Supplementary Table S1). The transduction of PHNs with rAAV2 and rAAV9 virions led to three fundamental findings: (1) for each target gene, a sgRNA molecule was identified that caused reduction of transcript levels, (2) rAAV9 virions were better suited than rAAV2 virions for delivering the cargo into PHNs as well as OHSCs, and (3) the knock-down effects were gene-specific. However, in comparison to controls that were conducted with scrambled sgRNA versions, the *hcn* transcript levels were reduced in neurons (~20–40% reduction). An explanation for this moderate efficiency at *hcn* gene loci might be imprecise assignment of the TSS regions, the most challenging part in designing CRISPRi experiments. Upon binding of the dSaCas9-KRAB-sgRNA complex to the target sequence, KRAB induces heterochromatin formation [47], and finally impairs RNA polymerase from initiating transcription. Several online tools are currently available to assist in identifying gene promoters [48]. To improve the CRISPRi efficacy for *hcn* transcript knock-down, one could re-examine the current TSS annotation with independent prediction algorithms to uncover additional and potentially favorable sgRNA target sites [48]. Alternatively, the TSS could be experimentally determined by molecular biological tools, like 5′ primer-extension or 5′ rapid extension of cDNA ends (RACE) [49,50]. However, these experiments were beyond the scope of the current investigation. Nevertheless, the CRISPRi approach demonstrated specific and reasonable *hcn* transcript knock-down. In comparison to conventional CRISPR/Cas experiments, CRISPRi might overcome potential complaints concerning off-target effects, due to enzymatically inactive dSaCas9 [39].

In comparison to the results that were obtained with CRISPRi, RNAi approaches resulted in robust knock-down of *hcn* transcript levels in recombinant cell lines, PHNs, as well as OHSCs. Interfering with transcript levels by RNAi strategies has the advantage that the target site(s) to which shRNA molecules can bind are less restricted. In principle, the entire primary transcript of a gene might serve as a template for shRNA binding [30]. For each *hcn* gene, we designed at least four individual shRNA molecules (Supplementary Table S1) and examined their efficacy and specificity in recombinant cell lines expressing homomeric HCN channels. For each *hcn* gene, we identified at least one shRNA molecule (see Table 3) that effectively reduced the expression of its target gene and did not cross-react with the other HCN channel-encoding genes. In transgenic cell-lines, transcript and protein levels were specifically reduced when compared to control values. With approximately 80% reduction, an impairment of *hcn2* and *hcn4* transcript levels in rAAV-transduced PHNs reached similar values to those that were determined in transgenic cell lines.

The main physiological question in this study was the dissection of the specific contribution of a single HCN isoform to the neuronal I_h_ current expressed in hippocampal pyramidal neurons. HCN channels play an important role in neuronal signaling and, here, we specifically examined the effects of HCN4 channel loss on basic electrophysiological properties of hippocampal neurons. As the basal transcript level of *hcn4* in hippocampal neurons is low when compared to *hcn1* and, especially, *hcn2* transcript levels, the HCN4 isoform might serve to diversify native I_h_ currents in heterotetrameric channels [9]. Indeed, electrophysiological recordings of PHNs showed that HCN4 has a substantial influence on I_h_ current activation potentials and kinetics, but not on basic neuronal properties, like resting membrane potential or input resistance, which is in contrast to the knock-out and knock-down of HCN1 (see Figures S5 and S6). Therefore, we suggest that the level of *hcn4* expression might diversify I_h_ current kinetics and activation potentials. By incorporating the subunit into functional heterotetrameric ion channels, the pronounced sensitivity of the HCN4 subunit to cyclic nucleotides might additionally modulate channel properties [6]. Notably, in a previous study, we have shown that a knock-down of HCN4 channel expression in the hippocampus of adult mice lead to a pronounced anxiogenic effect, which had not been observed, e.g., by HCN1 channel down-regulation [50]. In summary, our study has shown that RNAi-mediated knock-down of HCN channel expression in recombinant cell lines, PHNs, and OHSCs is efficient and specific. In comparison to transgenic approaches, like gene knock-out, conditional gene knock-out, or gene-editing by CRISPR/Cas9, the current experimental design is less time consuming as well as less prone to potential off-target effects or developmental failure. Furthermore, using rAAVs for cargo delivery to post-mitotic PHNs supports current ideas [43,44,51] to use viral transductions in basic and translational approaches.

## Figures and Tables

**Table 1:** Primary Antibodies Used for Immunocytochemistry. List of Primary Antibodies Applied for Immunocytochemistry. Abbreviations: gp, guinea pig; rb, rabbit; rt, rat; ch, chicken.

Antigen	Source	Dilution	Supplier
HCN1	gp	1:500	in house
HCN2	rb	1:500	in house
HCN4	rb	1:500	in house
HA	rt	1:100	Roche/Merck
GFP	ch	1:1000	chemicon (ab16901)
MAP 2	rb	1:1000	Synaptic Systems (188 002)
TOPRO-3		1:1000	Invitrogen (T3605)

**Table 2:** Secondary Antibodies Used for Immunocytochemistry. List of Secondary Antibodies Supplied for Immunocytochemistry. Abbreviations: gp, guinea pig; rb, rabbit; rt, rat; ch, chicken; dk, donkey.

Antibody	Source	Dilution	Supplier
α gp Cy3	dk	1:500	Dianova (706-165-148)
α rb Dy488	dk	1:500	Dianova (711-485-152)
α rt Cy5	dk	1:500	Dianova (712-175-153)
α rb Cy3	dk	1:500	Dianova (711-165-152)
α rt Dy488	gt	1:500	Invitrogen (A11006)
α ch Cy2	dk	1:200	Dianova (703-225-155)

**Table 3:** Primer Pairs Used for RT-qPCR analysis. Primer sequences (F, forward; R, reverse) and Amplicon Sizes are Based on Species-Specific cDNA Sequences. Amplicon Sizes of *gapdh* fragments are Indicated for Amplification on cDNA (150 bp) or on Genomic DNA (284 bp), thus allowing to monitor genomic contaminations.

Target	Primer Sequences	Amplicon Size (bp)
GAPDH	F: GGCATTGTGGAAGGGCTCATG	150/284
NM_008084.2	R: GCCCACAGCCTTGGCAGC	
HCN1	F: CTCAGTCTCTTGCGGTTATTACG	91
NM_010408.3	R: TGGCGAGGTCATAGGTCATG	
HCN2	F: ATCGCATAGGCAAGAAGAACTC	102
NM_008226.2	R: CAATCTCCTGGATGATGGCATT	
HCN4	F: GCATGATGCTTCTGCTGTGT	123
NM_001081192.1	R: GCTTCCCCCAGGAGTTATTC	
dSaCas9	F: CAGATTCAAGACCAGCGACTAC	103
HE980450.1	R: GTCGATGTAGGTGTCGATGAAG	
eGFP	F: GACGTAAACGGCCACAAGTTC	198
JQ064510.1	R: GAAGTCGTGCTGCTTCATGTG	

**Table 4:** Sequences and Positions of Functional sgRNAs and shRNAs. Sequences of Individual sgRNAs and Their Target Positions Relative to the Transcriptional Start Site (TSS) and Sequences of Individual shRNAs and Their Target Positions Relative to the ATG Start Codon are Summarized. Target Sequences of sgRNAs are Based on the Predicted TSS from the Eukaryotic Promoter Database (EPD) [28]. Target Sequences for shRNAs are Based on the Murine mRNA Sequences NM_010408.3 for HCN1, NM_008226.2 for HCN2, and NM_001081192.1 for HCN4.

Target Gene	Name	Sequence	Position (bp)
HCN1	sg1	F: CTCCGCGTCCAACAGCCGCGAC R: GTCGCGGCTGTTGGACGCGGAG	227–248
HCN2	sg2	F: TCGCACCCGGAGTCGGCGGAC R: GTCCGCCGACTCCGGGTGCGA	162–182
HCN4	sg4	F: GTAGAGGAGGCAAAGCGAGAAC R: GTTCTCGCTTTGCCTCCTCTAC	135–159
	sgScr	F: CAACAAGATGAAGAGCACCAA R: TTGGTGCTCTTCATCTTGTTG	
HCN1	sh1	F: CCTCCAATCAACTATCCTCAA R: TTGAGGATAGTTGATTGGAGG	1876–1896
HCN2	sh2	F: CCATGCTGACAAAGCTCAAAT R: TTTGAGCTTTGTCAGCATGG	1583–1603
HCN4	sh4	F: CTCCAAACTGCCGTCTAATTT R: AAATTAGACGGCAGTTTGGAG	3582–3602
	shScr	F: CAACAAGATGAAGAGCACCAA R: TTGGTGCTCTTCATCTTGTTG	

## Data Availability

The data and materials presented in this study are available on request from the corresponding author.

## Supplementary Materials

The following are available online at https://www.mdpi.com/2073-4409/10/2/324/s1, Figure S1: rAAV-mediated gene knock-down by CRISPRi and RNAi, Figure S2: sgRNAs guide dSaCas9 to the promoter of target genes, Figure S3: sgRNAs guide dSaCas9 to the promoter of target genes, Figure S4: HCN4 channel knock-down has no effect on basic parameters of PHNs, Figure S5: Immunohistochemistry and I_h_-current comparison of HCN1 knock-out vs knock-down PHNs, Figure S6: Effects of HCN-channel knock-out and knock-down on I_h_-current activation and sag potential, Table S1: Sequences and positions of all tested sgRNAs and shRNAs, Table S2: Electrophysiological properties of hippocampal neurons and HEK293 cells.
