# Supplementary material for: AAV-Mediated CRISPRi and RNAi Based Gene Silencing in Mouse Hippocampal Neurons
Source: Cells. 2021 Feb 4;10(2):324. doi: 10.3390/cells10020324 (PMC7915209; doi:10.3390/cells10020324)
Supplement: Supplementary file 1 [file cells-10-00324-s001.pdf]

AAV-Mediated CRISPRi and RNAi Based Gene Silencing in Mouse Hippocampal Neurons

Matthias Deutsch, Anne Günther, Rodrigo Lerchundi, Christine R. Rose, Sabine Balfanz, Arnd Baumann

Supplementary Material

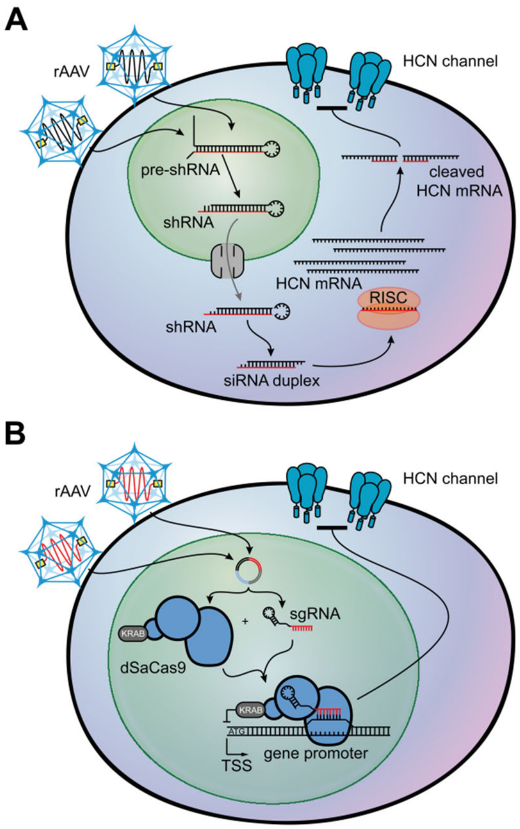

Figure S1. rAAV-mediated gene knock-down by CRISPRi and RNAi. (**A**) Schematic representation of the RNA silencing mechanism mediated by RNA interference. rAAV, recombinant adeno-associated virus; shRNA, short-hairpin RNA; siRNA, short-interfering RNA; mRNA, messenger RNA; RISC, RNA-induced silencing complex; HCN, hyperpolarization-activated cyclic nucleotide-gated cation channel. (**B**) Schematic representation of the gene inactivation mechanism mediated by CRISPR interference. rAAV recombinant adeno-associated virus; dSaCas9, nuclease deficient *Staphylococcus aureus* Cas9; HCN, hyperpolarization-activated cyclic nucleotide-gated cation channel; sgRNA, short-guidance RNA; ATG, translational start codon; TSS, transcriptional start site; KRAB, Krüppel-associated box.

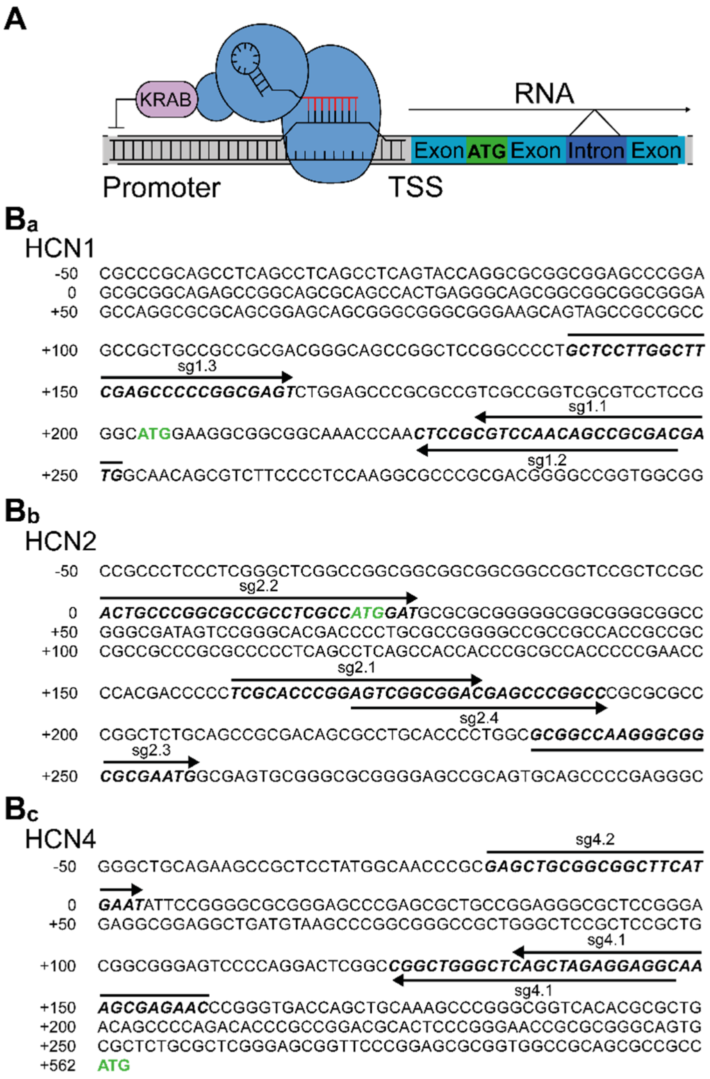

Figure S2. sgRNAs guide dSaCas9 to the promoter of target genes. (**A**) Schematic representation illustrating the guidance and binding of dSaCas9 to the promoter of a target gene. (**B**) Sequences of individual sgRNAs and their target positions relative to the transcriptional start site (TSS) for (**B$_a$**) HCN1, (**B$_b$**) HCN2, and (**B$_c$**) HCN4.

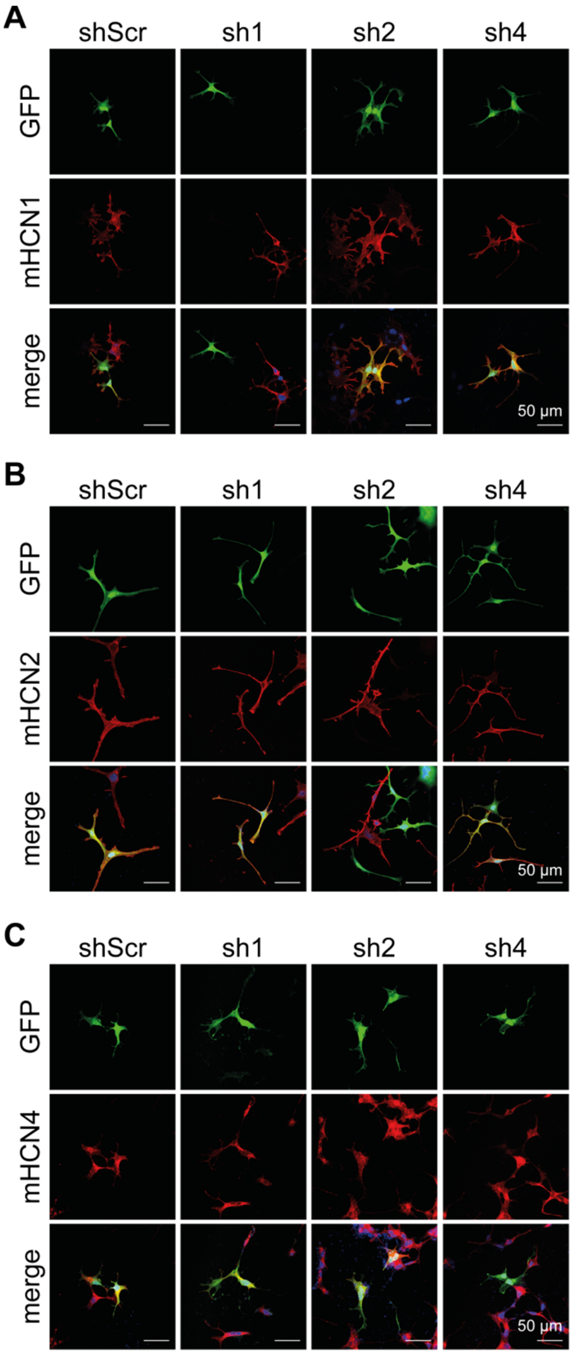

Figure S3. sgRNAs guide dSaCas9 to the promoter of target genes. Immunofluorescence images showing the expression shRNAs (shScr, sh1, sh2 and sh4) in HEK293 cells constitutively expressing (**A**) HCN1, (**B**) HCN2, and (**C**) HCN4 proteins. Immunostainings were used to calculate Pearson's R values for colocalization analysis of HCN channel knock-down.

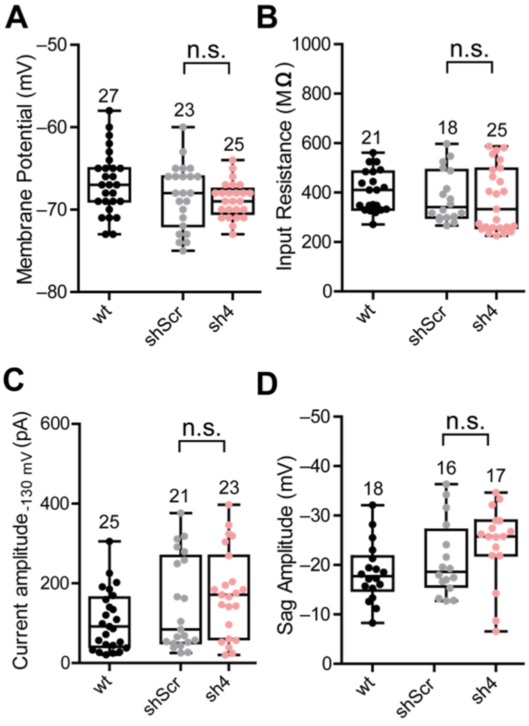

Figure S4: HCN4 channel knock-down has no effect on basic parameters of PHNs. (**A-D**) Whole-cell patch-clamp recordings from wildtype (non-transduced) or rAAV9-transduced eGFP positive primary hippocampal neurons. (**A**) Knock-down of HCN-channel subunit 4 (sh4) induced no change in the resting membrane potential compared to neurons expressing a scrambled control construct (shScr). (**B**) Input resistances to 10 mV hyperpolarizing voltage pulses of sh4-transduced neurons increased compared to shScr-transduced neurons. (**C**) No changes in the I_h-current amplitude upon a hyperpolarizing step pulse of -130 mV, or (**D**) in the sag-potential amplitude upon a current step which hyperpolarizes the neuron to a membrane potential of -130 mV, was observed in sh4- or shScr-transduced neurons. Results are depicted as boxplots. Statistical significance was assessed using the unpaired two-tailed Student´s *t* test.

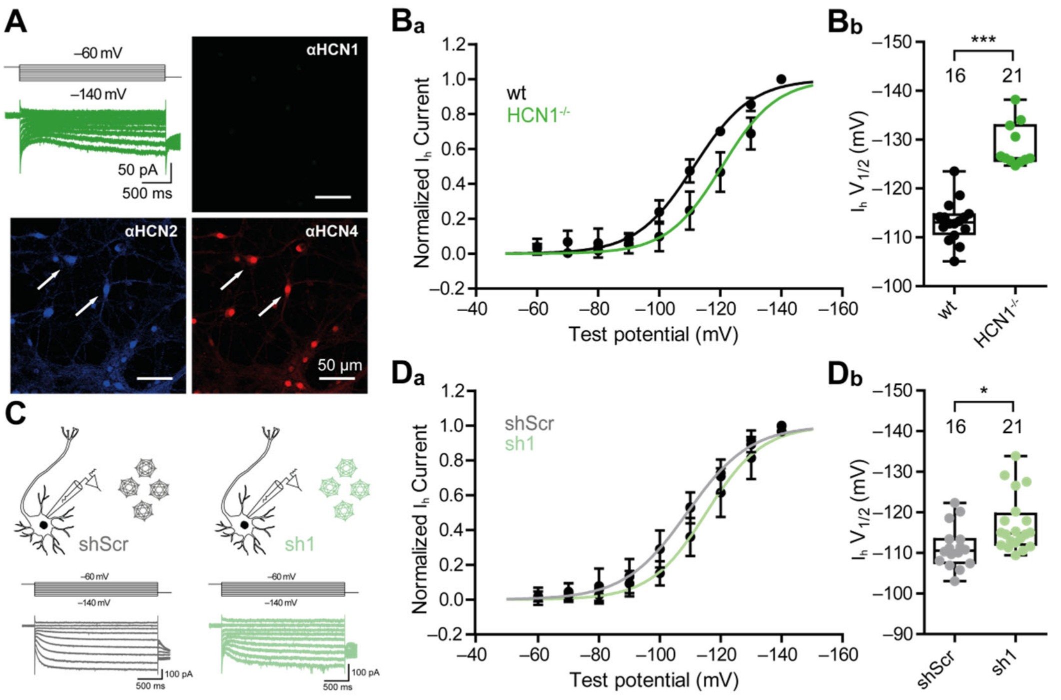

Figure S5: Immunohistochemistry and I_h-current comparison of HCN1 knock-out vs knock-down PHNs. (**A**) Representative voltage stimulation protocol and corresponding current traces of whole-cell patch-clamp recordings of PHNs derived from HCN1 channel knock-out mouse (HCN1$^{-/-}$) 15 div. Representative immunofluorescent images of the same neurons showing expression of HCN-channel isoforms 1 (green), 2 (blue) and 4 (red). (**B**$_a$) Current-voltage relationships recorded from PHNs of wildtype and HCN1$^{-/-}$ mice. (**B**$_b$) Half-maximal activation voltages of recordings from PHNs of wildtype and HCN1$^{-/-}$ mice. Results are depicted as boxplots. (**C**) Representative voltage stimulation protocols and corresponding current traces of whole-cell patch-clamp recordings derived from rAAV9-transduced PHNs expressing shScr (control) or sh1 (HCN1 channel knock-down). (**D**$_a$) Current-voltage relationships recorded from rAAV9-transduced, shScr or sh1 expressing PHNs. (**D**$_b$) Half-maximal activation voltages from rAAV9-transduced, shScr or sh1 expressing PHNs. Results are depicted as boxplots. Statistical significance was assessed using the unpaired two- tailed Student´s t test, *$p<0.05$, ***$p<0.001$. In both, primary hippocampal neurons from HCN1$^{-/-}$ knock-out as well as sh1-treated (= HCN1 knock-down) neurons, half-maximal activation voltages are shifted to more negative values.

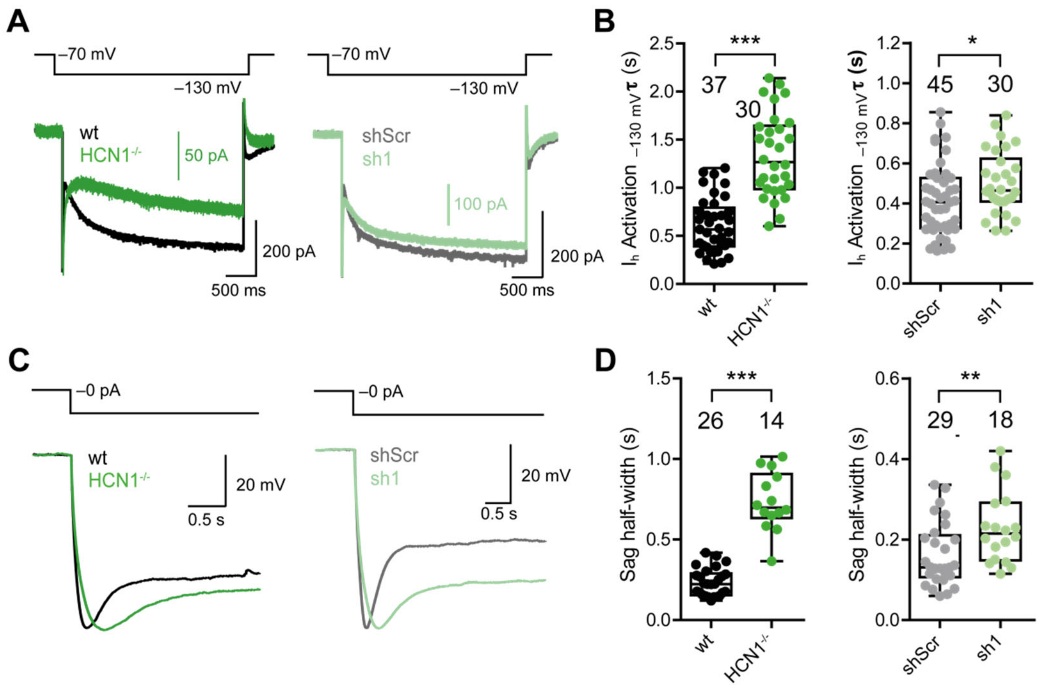

Figure S6: Effects of HCN-channel knock-out and knock-down on I_h-current activation and sag potential. (**A**) Representative voltage stimulation and corresponding current traces of whole-cell patch-clamp recordings derived from wt, HCN1$^{-/-}$, shScr-treated, or sh1-treated PHNs. (**B**) Current amplitudes of PHNs from wt and HCN1$^{-/-}$ mice (left), and of rAAV9-shScr and -sh1 transduced eGFP-positive PHNs (right). Current amplitudes were measured from the difference of the instantaneous current and the steady-state current to the current response of a hyperpolarizing pulse (from -70 mV to -130 mV). (**C**) Representative current stimulation and corresponding voltage traces of whole-cell patch-clamp recordings derived from from wt, HCN1$^{-/-}$ mice, shScr-treated, and sh1-treated PHNs. (**D**) Sag potential half-widths of PHNs from wt and HCN1$^{-/-}$ mice (left), and of rAAV9-shScr and -sh1 transduced eGFP-positive PHNs (right). Sag potentials were evoked by current pulses hyperpolarizing the membrane potential to -130 mV. Results are depicted as boxplots. Statistical significance was assessed using the unpaired two-tailed Student´s t test, *p<0.05, **p<0.01, ***p<0.001.

Table S1: Sequences and positions of all tested sgRNAs and shRNAs. Sequences of individual sgRNAs and their target positions relative to the transcriptional start site (TSS) and sequences of individual shRNAs and their target positions relative to the ATG start codon are summarized. Target sequences of sgRNAs are based on the predicted TSS from the Eukaryotic Promoter Database (EPD). Target sequences for shRNAs are based on the murine mRNA sequences NM_010408.3 for HCN1, NM_008226.2 for HCN2, and NM_001081192.1 for HCN4.

target gene	name	sequence	position (bp)
HCN1	sg1.1	F: CGTCCAACAGCCGCGACGATGC	232–252
		R: GCATCGTCGCGGCTGTTGGACG	
HCN1	sg1.2	F: CTCCGCGTCCAACAGCCGCGAC	227–248
		R: GTCGCGGCTGTTGGACGCGGAG	
HCN1	sg1.3	F: CTCCTTGGCTTCGAGCCCCCGGCGAGT	139–166
		R:ACTCGCCGGGGGCTCGAAGCCAAGGAGC	

HCN2	sg2.1	F: TCGCACCCGGAGTCGGCGGAC	162–182
		R: GTCCGCCGACTCCGGGTGCGA	
HCN2	sg2.2	F: GACTGCCCGGCGCCGCCTCGCCATGGAT	0–27
		R: ATCCATGGCGAGGCGGCGCCGGGCAGTC	
HCN2	sg2.3	F: GCGGCCAAGGGCGGCGCGAATG	238–259
		R: CATTCGCGCCGCCCTTGGCCGC	
HCN2	sg2.4	F: AGTCGGCGGACGAGCCCGGCC	173–193
		R: GGCCGGGCTCGTCCGCCGACT	
HCN4	sg4.1	F: GTAGAGGAGGCAAAGCGAGAAC	139–159
		R: GTTCTCGCTTTGCCTCCTCTAC	
HCN4	sg4.2	F: GAGCTGCGGCGGCTTCATGAAT	-17– 4
		R: ATTCATGAAGCCGCCGCAGCTC	
HCN4	sg4.3	F: CGGCTGGGCTCAGCTAGAGGC	125–144
		R: GCCTCTAGCTGAGCCCAGCCG	
	sgScr	F: CAACAAGATGAAGAGCACCAA	
		R: TTGGTGCTCTTCATCTTGTTG	
HCN1	sh1.1	F: GTGGCCTACATGCAAATGTAA	2862–2882
		R: TTACATTTGCATGTAGGCCAC	
HCN1	sh1.2	F: GCTGGGTTTCTCTGAATGAAA	957–977
		R: TTTCATTCAGAGAAACCCAGC	
HCN1	sh1.3	F: CCTCCAATCAACTATCCTCAA	1876–1896
		R: TTGAGGATAGTTGATTGGAGG	
HCN1	sh1.4	F: GCGCCAGAAGATACATGATTA	1252–1272
		R: TAATCATGTATCTTCTGGCGC	
HCN1	sh1.5	F: GCACTTCGTATCGTGAGGTTT	728–748
		R: AAACCTCACGATACGAAGTGC	
HCN2	sh2.1	F: CCATGCTGACAAAGCTCAAAT	1583–1603
		R: TTTGAGCTTTGTCAGCATGG	
HCN2	sh2.2	F: CTGTTGTTCATGGTGGGAAAT	574–594
		R: ATTTCCCACCATGAACAACAG	
HCN2	sh2.3	F: GCATTGTTATTGAGGACAACA	713–733
		R: TGTTGTCCTCAATAACAATGC	
HCN2	sh2.4	F: CCGGCATTGTTATTGAGGACA	716–736
		R: TGTCCTCAATAACAATGCCGG	
HCN4	sh4.1	F: GAGAGGAGATCATCAACTTTA	1733–1753
		R: TAAAGTTGATGATCTCCTCTC	
HCN4	sh4.2	F: CTCCAAACTGCCGTCTAATTT	3582 3602
		R: AAATTAGACGGCAGTTTGGAG	
HCN4	sh4.3	F: AGCGCATCCATGACTACTATG	1646–1666
		R: CATAGTAGTCATGGATGCGCT	
HCN4	sh4.4	F: AGCGTCAGAGCGGATACTTAT	2014–2034
		R: ATAAGTATCCGCTCTGACGCT	
HCN4	sh4.5	F: GAAGACATCCTCAGGTTCTTT	3453–3473
		R: AAAGAACCTGAGGATGTCTTC	
	shScr	F: CAACAAGATGAAGAGCACCAA	
		R: TTGGTGCTCTTCATCTTGTTG	

Table S2: Electrophysiological properties of hippocampal neurons and HEK293 cells. Electrophysiological parameters were measured using whole-cell voltage clamp and current clamp recordings. For a detailed transcription see "Materials and Methods" section. Numbers of cells tested are shown in parentheses. Parameters are depicted as mean ± standard deviation.

	**E_m** <br>**(mV)**	**R_{in}** <br>**(MΩ)**	**I_h (-130 mV)** <br>**(pA)**	**Sag (-130 mV)** <br>**(mV)**	**I_h $V_{1/2}$** <br>**(mV)**	**I_h** τ (-130 mV) <br>**(ms)**	**Sag** τ (-130 mV) <br>**(ms)**
**WT**	**-66.74** ± 3.819 **(27)**	**404.7** ± 82.66 **(21)**	**106.3** ± 75.33 **(25)**	**18.46** ± 5.964 **(18)**	**-111.7** ± 3 **(7)**	**-647.2** ± 327.5 **(13)**	**252.2** ± 101 **(13)**
**HCN1$^{-/-}$**	**-71.0** ± 2.9 **(35)**	**538.6** ± 115.0 **(35)**	**34.28** ± 38.23 **(40)**		**-128.8** ± 4.47 **(21)**	**1341** ± 428 **(30)**	**778** ± 126 **(14)**
**shScr**	**-68.35** ± 3.9 **(23)**	**382.7** ± 104.2 **(18)**	**146.6** ± 117.4 **(21)**	**21.13** ± 7.63 **(16)**	**-111.3** ± 3.84 **(9)**	**382** ± 181.1 **(13)**	**143.2** ± 76.27 **(13)**
**sh1**	**-71.0** ± 3.29 **(27)**	**457.5** ± 125.1 **(28)**	**-107.0** ± 74.09 **(34)**		**-117.1** ± 6.9 **(21)**	**503** ± 156 **(30)**	**229** ± 38 **(18)**
**sh4**	-68.8 ± 2.16 **(25)**	**372.7** ± 130.4 **(25)**	**174.7** ± 109.7 **(23)**	**24.28** ± 7.86 **(17)**	**-106.4** ± 3.42 **(15)**	**238.1** ± 131.6 **(16)**	**92.06** ± 23.82 **(16)**
**HEK293-HCN1**					**-96.14** ± 1.63 **(7)**	**71.6** ± 43.9 **(19)**	**37.9** ± 21 **(9)**
**HEK293-HCN2**					**-108.5** ± 1.3 **(8)**	**269.7** ± 97 **(26)**	**83.29** ± 38.9 **(14)**
**HEK293-HCN4**					**-119.3** ± 5.86 **(4)**	**934.4** ± 183.2 **(18)**	**298.6** ± 56.47 **(10)**
